# Posttranscriptional Regulation of HIV-1 Gene Expression during Replication and Reactivation from Latency by Nuclear Matrix Protein MATR3

**DOI:** 10.1128/mBio.02158-18

**Published:** 2018-11-13

**Authors:** Ambra Sarracino, Lavina Gharu, Anna Kula, Alexander O. Pasternak, Veronique Avettand-Fenoel, Christine Rouzioux, Maryana Bardina, Stéphane De Wit, Monsef Benkirane, Ben Berkhout, Carine Van Lint, Alessandro Marcello

**Affiliations:** a Laboratory of Molecular Virology, The International Center for Genetic Engineering and Biotechnology (ICGEB), Trieste, Italy; b Institute for Molecular Biology and Medicine (IBMM), University of Brussels (ULB), Brussels, Belgium; c Malopolska Centre of Biotechnology, Jagiellonian University, Krakow, Poland; d Laboratory of Experimental Virology, Department of Medical Microbiology, Academic Medical Center of the University of Amsterdam, Amsterdam, The Netherlands; e Service de Virologie, Hôpital Necker-Enfants-Malades, Université Paris-Descartes, EA7327, AP-HP, Paris, France; f Service des Maladies Infectieuses, CHU St-Pierre, Université Libre de Bruxelles (ULB), Brussels, Belgium; g Laboratory of Molecular Virology, Institute of Human Genetics, Montpellier, France; University College London; Columbia University

**Keywords:** HIV-1, latency, posttranscription, MATR3, RNA, latency-reverting agents, LRA, shock and kill, reservoir, RNA binding proteins, human immunodeficiency virus, posttranscriptional control mechanisms

## Abstract

Posttranscriptional regulation of HIV-1 replication is finely controlled by viral and host factors. Among the former, Rev controls the export of partially spliced and unspliced viral RNAs from the nucleus and their translation in the cytoplasm or incorporation into new virions as genomic viral RNA. To investigate the functional role of the Rev cofactor MATR3 in the context of HIV infection, we modulated its expression in Jurkat cells and primary peripheral blood lymphocytes (PBLs). We confirmed that MATR3 is a positive regulator of HIV-1 acting at a posttranscriptional level. By applying the same approach to J-lat cells, a well-established model for the study of HIV-1 latency, we observed that MATR3 depletion did not affect transcriptional reactivation of the integrated provirus, but caused a reduction of Gag production. Following these observations, we hypothesized that MATR3 could be involved in the establishment of HIV-1 posttranscriptional latency. Indeed, mechanisms acting at the posttranscriptional level have been greatly overlooked in favor of transcriptional pathways. MATR3 was almost undetectable in resting PBLs, but could be promptly upregulated upon cellular stimulation with PHA. However, HIV latency-reversing agents were poor inducers of MATR3 levels, providing a rationale for their inability to fully reactivate the virus. These data have been confirmed *ex vivo* in cells derived from patients under suppressive ART. Finally, in the context of MATR3-depleted J-lat cells, impaired reactivation by SAHA could be fully rescued by MATR3 reconstitution, demonstrating a direct role of MATR3 in the posttranscriptional regulation of HIV-1 latency.

## Abstract (precis)

The life cycle of HIV-1 requires integration of a DNA copy into the genome of the host cell. Transcription of the viral genes generates RNAs that are exported to the cytoplasm with the contribution of viral and cellular factors to get translated or incorporated in the newly synthesized virions. It has been observed that highly effective antiretroviral therapy, which is able to reduce circulating virus to undetectable levels, cannot fully eradicate the virus from cellular reservoirs that harbor a transcriptionally latent provirus. Thus, persistence of latently infected cells is the major barrier to a cure for HIV-1 infection. In order to purge these reservoirs of latently infected cells, it has been proposed to activate transcription to stimulate the virus to complete its life cycle. This strategy is believed to unmask these reservoirs, making them vulnerable to the immune system. However, limited successes of this approach may indicate additional posttranscriptional restrictions that need to be overcome for full virus reactivation. In this work we identify the cellular protein MATR3 as an essential cofactor of viral RNA processing. Reactivation of HIV-1 transcription *per se* is not sufficient to allow completion of a full life cycle of the virus if MATR3 is depleted. Furthermore, MATR3 is poorly expressed in quiescent CD4^+^ T lymphocytes that are the major reservoir of latent HIV-1. Cells derived from aviremic HIV-1 patients under antiretroviral therapy didn’t express MATR3, and most importantly, latency-reversing agents proposed for the rescue of latent provirus were ineffective for MATR3 upregulation. To conclude, our work identifies a cellular factor required for full HIV-1 reactivation and points to the revision of the current strategies for purging viral reservoirs that focus only on transcription.

## INTRODUCTION

Combination antiretroviral therapy (cART) has successfully reduced human immunodeficiency virus type 1 (HIV-1)-related disease (1). However, effective therapy requires a lifelong regimen of cART to maintain undetectable viremia. Blood and tissue reservoirs that are insensitive to drug treatment maintain a source of virus, which inevitably rebounds following interruption of therapy. The most studied reservoirs are quiescent memory T cells that harbor an integrated transcriptionally silent but replication-competent cDNA copy of the virus in their genomes. Experimental virus eradication therapies called “shock and kill” strategies are being proposed to awake dormant viruses in the latent reservoir. Virus-producing cells would then be susceptible to HIV-induced killing and clearance by the immune system (2, 3). Current regimens for HIV reactivation are based on drugs known as latency-reversing agents (LRAs) that induce transcriptional activation of the provirus (1, 4). Despite successes *ex vivo*, clinical studies of single LRAs have achieved only limited success in the depletion of the reservoir (5, 6). Failure to completely eradicate viral infection with LRAs *in vivo* might be related to poor immunity toward reactivated cells, and studies are under way using combination of LRAs associated with strategies to improve the HIV-specific immune response (7). Alternatively, some cells of the reservoir could maintain a latent replication-competent provirus insensitive to LRA either because of insufficient penetrance of the drug or because some posttranscriptional block remains insensitive to LRAs. Despite few reports, the latter aspect has been greatly overlooked and may severely impact successful LRA approaches (8).

HIV transcription is extremely efficient once the viral Tat transactivator is expressed, generating a positive feedback loop (9, 10). Nascent viral RNA is then processed by the cellular machinery to generate a number of spliced, partially spliced, or unspliced (genomic) products. While fully spliced viral RNAs follow the classical nuclear export pathway, partially spliced and unspliced viral RNAs follow an alternative route mediated by the viral protein Rev (11). Rev binds the Rev-responsive element (RRE) and promotes the export of RRE-containing viral RNAs from the nucleus through interaction with Exportin 1 (XPO1) (12–14). We previously affinity purified the HIV ribonucleoprotein complex in the cell nucleus (15). One of the proteins identified by mass spectrometry was the matrix-associated RNA binding protein Matrin 3 (MATR3), which was shown to interact with Rev through RRE and was required for Rev-mediated export of RRE-containing HIV-1 RNAs (15, 16). MATR3 forms a complex with another protein identified in the screen: the polypyrimidine tract-binding protein-associated binding factor PSF (splicing factor proline and glutamine rich, SFPQ), which was already implicated in Rev-mediated export of HIV-1 RNAs (17). We could demonstrate that, while Rev and PSF bind the viral pre-mRNA at the site of viral transcription, MATR3 interacts at a subsequent step involved in nuclear export. Therefore, PSF and MATR3 define a nuclear pathway for RRE-containing HIV-1 RNAs that is hijacked by the viral Rev protein (18).

In this work we provide evidence of a critical role for MATR3 in acute HIV-1 infection and reactivation from latency. Data from CD8^+^ depleted peripheral blood mononuclear cells (PBMC) from aviremic cART patients indicate an intriguing correlation between low levels of MATR3/PSF and poor performance of LRAs *ex vivo*. We believe these data will stimulate research toward the definition of posttranscriptional pathways in the reactivation from latency and their exploitation for new-generation LRAs.

## RESULTS

### MATR3 depletion inhibits nuclear export of HIV-1 unspliced RNA in lymphocytes.

**FIG 1:**
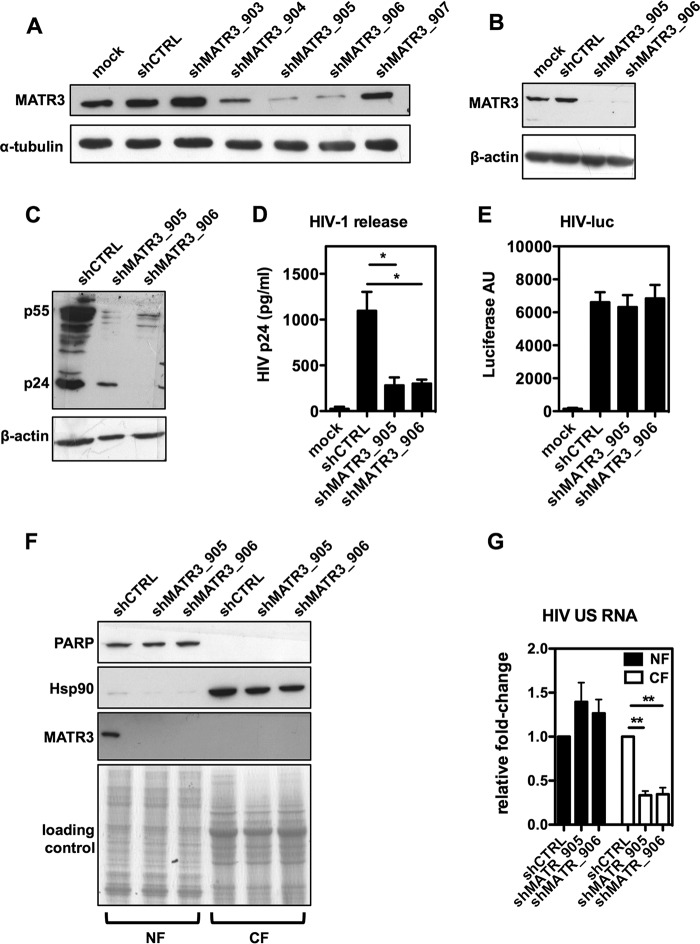
Depletion of MATR3 inhibits HIV-1 Gag expression and nuclear export of unspliced RNA. (A) Optimization of shMATR3 lentivectors. HeLa cells were transduced with lentivectors targeting MATR3 (shMATR3) or with control shRNA (shCTRL). Three days after puromycin selection, MATR3-depleted cells were harvested for immunoblotting. α-Tubulin is the protein loading control. (B) Depletion of MATR3 in Jurkat cells. Lentivectors for MATR3 depletion were used to transduce Jurkat cells, which were processed as indicated above. β-Actin is the loading control. (C) MATR3 knockdown leads to the decrease of HIV-1 Gag expression. Jurkat cells were transduced with shMATR3. Three days after puromycin selection, MATR3-depleted cells were infected with replication-competent HIV-1 NL4.3 virus and harvested 48 h postinfection for immunoblotting. β-Actin is the protein loading control. (D) MATR3 depletion leads to the decrease in the extracellular release of HIV-1 p24. MATR3-depleted Jurkat cells generated as described above were infected with HIV-1 pNL-4.3 virus, and supernatant was harvested 48 h postinfection. Virion production in the supernatant was quantified by HIV-1 p24 ELISA. Average values of three independent experiments are shown, with standard deviations (SD) and *P* values as described in Materials and Methods. (E) HIV-1 LTR-driven transcription is not affected by MATR3 knockdown. MATR3-depleted Jurkat cells generated as described above were infected with HIV-1 pNL-4.3 luciferase virus (pNL-4.3-R-E-luc) pseudotyped with the VSV-G envelope. Luciferase was measured 48 h postinfection. Relative luciferase expression was normalized to total protein levels as measured by Bradford assay. The results of three independent experiments are shown as mean values ± SD. (F) Nucleocytoplasmic fractionation of Jurkat cells. Following shMATR3 transduction and infection as described above, Jurkat cells were subjected to nucleocytoplasmic fractionation. The cytoplasmic marker protein Hsp90 was detected only in the cytoplasm, while the nuclear marker PARP was detected only in the nuclear fraction. MATR3 was detected only in the nucleus of shCTRL-treated cells and depleted from the nuclear extracts of cells treated with shMATR3_905 and shMATR3_906. The Coomassie blue-stained gel was used as the loading control. (G) HIV-1 unspliced RNA levels are modulated by MATR3. Quantitative analysis of unspliced HIV-1 RNA levels in Jurkat cells treated as above. Unspliced (US) RNA levels were analyzed by quantitative real-time PCR on nuclear (NF) and cytoplasmic (CF) fractions. Data were normalized to GAPDH mRNA expression and presented as fold changes compared to shCTRL. Average values of triplicate independent experiments are shown, with standard deviations (SD) and *P* values as described in Materials and Methods.

To investigate the role of MATR3 in HIV-1 replication, we needed a stable depletion of MATR3 in CD4^+^ T lymphocytes. To this end we identified two potent shRNAs targeting MATR3, shMATR3_905 and shMATR3_906 (Fig. 1A). Depletion of MATR3 was previously shown to affect Gag expression and the Rev-mediated nuclear export of unspliced viral RNA (15, 18). Consistently, infection of Jurkat cells depleted of MATR3 (Fig. 1B) with HIV-1 NL4.3 results in reduced intracellular expression of Gag as shown in Fig. 1C, corresponding to a marked decrease of extracellular p24 (Fig. 1D). To rule out a transcriptional effect of MATR3 depletion, Jurkat cells were infected with equal amounts of VSV-G pseudotyped, firefly luciferase-expressing HIV-1 (NL-4.3-R-E-luc) capable of a single cycle of infection. The luciferase reporter that is placed in the *nef* gene is expressed from a spliced transcript driven by the 5′ LTR. As shown in Fig. 1E, luciferase activity was not affected by shMATR3 depletion. To pinpoint the step at which HIV-1 replication was affected, we measured transcript levels in the nucleus and cytoplasm of infected cells depleted of MATR3. To check for the quality of nuclear and cytoplasmic fractionation and shRNA-mediated knockdown of MATR3, a routine immunoblot was performed for Hsp90 and PARP, which are the controls for cytoplasmic and nuclear fractions, respectively. Coomassie blue-stained SDS-PAGE was used as loading control (Fig. 1F). MATR3 could be recovered only from the nuclear fraction, as previously observed, and was greatly depleted by shRNA treatment. qRT-PCR was performed to analyze HIV unspliced RNA levels. We found that the levels of unspliced mRNA slightly increased in the nuclear fraction, but decreased significantly in the cytoplasmic fraction of MATR3-depleted cells (Fig. 1G). These results, obtained in the context of acute infection of CD4^+^ T lymphocytes with a replication-competent virus, further strengthen and extend our previous observation that MATR3 selectively acts on the Rev-dependent nuclear export of unspliced viral RNA.

### MATR3 depletion inhibits HIV-1 replication in lymphocytes.

**FIG 2:**
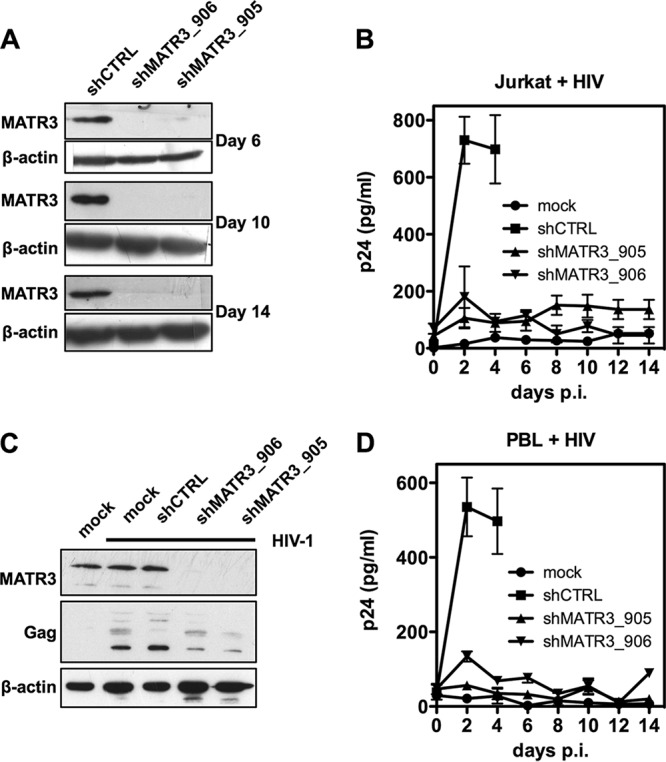
MATR3 depletion inhibits HIV-1 replication. (A) Long-term depletion of MATR3 in Jurkat cells. Western blot analysis of MATR3 after transduction at different time points. β-Actin is the protein loading control. (B) Impaired HIV-1 replication in MATR3-depleted Jurkat cells. Jurkat cells depleted for MATR3 as described were infected with replication-competent HIV-1 pNL-4.3 virus, and supernatant was harvested after every 2 days to monitor HIV replication. Virion production in the supernatant was quantified by HIV-1 p24 ELISA (shCTRL cells are shown only until peak p24, then the signal decreased due to cytotoxic effects of infection). The results of two independent experiments are shown as mean values ± SD. (C) MATR3 knockdown in peripheral blood lymphocytes. Primary human peripheral blood lymphocytes (PBLs) were transduced with shMATR3. Four days posttransduction, MATR3-depleted cells were harvested for immunoblotting against MATR3 or Gag as indicated. β-Actin is the protein loading control. (D) MATR3 depletion inhibits HIV-1 replication in primary human peripheral blood lymphocytes. MATR3-depleted PBLs, generated as described above, were infected with replication-competent HIV-1 pNL-4.3 virus. The supernatant was harvested every 2 days to monitor HIV replication. Virion production in the supernatant was quantified by HIV-1 p24 ELISA. The results of two independent experiments from different donors are shown as mean values ± SD.

We then proceeded to examine the replication kinetics of HIV-1 when MATR3 is depleted. At 24 hours after transduction with shRNAs, Jurkat cells were cultured in RPMI containing puromycin for 72 h and then left without selection. Immunoblotting showed a high level of depletion maintained for more than 2 weeks (Fig. 2A), and viability assays showed lack of cytotoxicity (not shown). Infection with HIV-1 NL4.3 resulted in the release of virions from day 2 in shCTRL cells, while a robust increase of p24 could not be detected in shMATR3 cells up to 14 days postinfection (Fig. 2B).

To extend this observation, primary peripheral blood lymphocytes (PBLs) from healthy donors were activated with PHA/IL-2, transduced with shRNA, and infected with HIV-1 as described above. Figure 2C and D show the efficient depletion of MATR3, the corresponding decrease of intracellular Gag, and the inhibition of HIV-1 NL4.3 replication.

These results demonstrate that depletion of MATR3 affects acute HIV-1 infection of CD4^+^ T lymphocytes.

### Ectopic MATR3 increases viral replication in lymphocytes.

**FIG 3:**
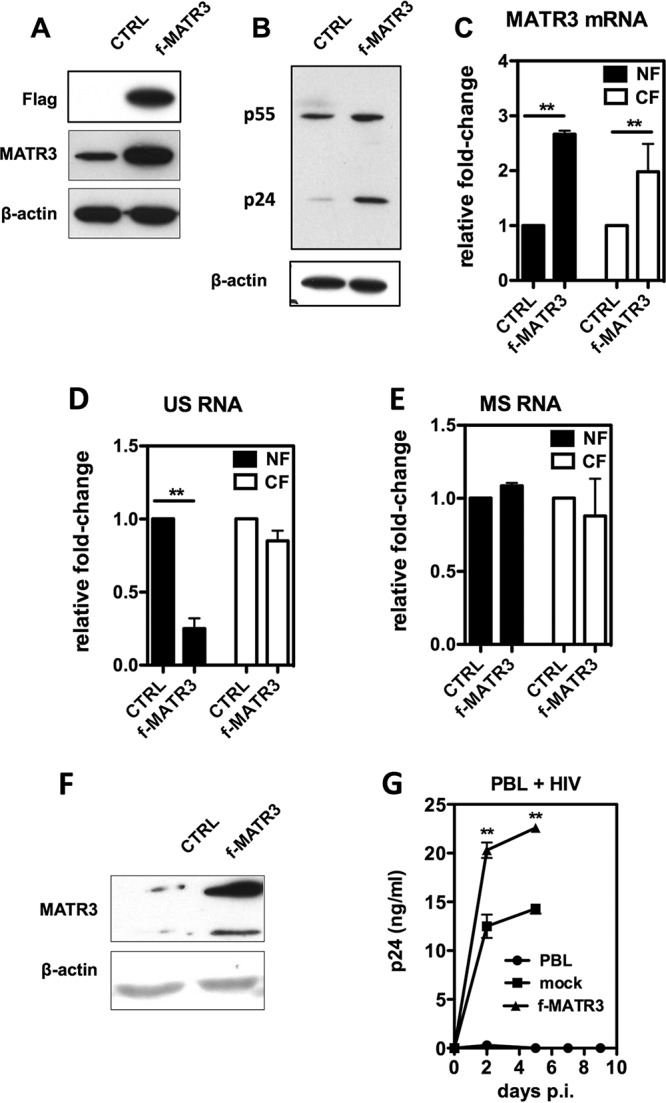
Ectopic MATR3 promotes HIV-1 replication. (A) Overexpression of MATR3 in Jurkat cells. Flag-tagged MATR3 (f-MATR3) was overexpressed in Jurkat cells following transduction with a lentivector. Immunoblot with the indicated antibodies was performed following blasticidin selection. β-Actin is the protein loading control. (B) Overexpression of MATR3 leads to an increase of Gag expression. Jurkat cells were transduced with f-MATR3 or with a control lentivector. Five days after blasticidin selection, cells were infected with replication-competent HIV-1 pNL-4.3 virus and harvested 48 h postinfection for immunoblotting. β-Actin is the protein loading control. (C) MATR3 RNA is increased both in the nucleus (NF) and in the cytoplasm (CF). Nucleocytoplasmic fractionation of Jurkat cells was performed as described in Materials and Methods. Quantification of MATR3 mRNA normalized to GAPDH expression is presented as fold changes compared to mock-transduced cells. Average values of duplicate independent experiments are shown, with standard deviations (SD) and *P* values as described in Materials and Methods. (D) HIV-1 unspliced RNAs decrease in the nucleus when MATR3 is overexpressed. Quantitative analysis of RNA levels was performed as described above. (E) HIV-1 multiply spliced RNAs are not affected when MATR3 is overexpressed. Quantitative analysis of RNA levels was performed as described above. (F) Ectopic f-MATR3 expression in PBLs. Lentivectors for the expression of f-MATR3 were used to transduce PBLs. Cells were then lysed and blotted for MATR3 and β-actin as loading control. (G) Ectopic f-MATR3 expression enhances HIV-1 replication in PBLs. PBLs expressing f-MATR3 were infected with replication-competent HIV-1 NL4.3 virus. The supernatant was harvested every 2 days to monitor HIV replication. Virion production in the supernatant was quantified by HIV-1 p24 ELISA. The results of two independent experiments are shown as mean values ± SD.

Jurkat cells were transduced with a lentiviral vector expressing flag-tagged MATR3, which resulted in higher MATR3 protein levels (Fig. 3A). Infection of these cells with HIV-1 NL4.3 showed an increased intracellular expression of Gag (Fig. 3B). Nucleocytoplasmic fractionation of infected Jurkat cells overexpressing f-MATR3 (Fig. 3C) resulted in a major decrease of US HIV-1 RNA in the nuclear fraction (Fig. 3D), while MS RNAs were not affected (Fig. 3E). Decrease of US RNA in the nucleus is compatible with increased export mediated by MATR3, while steady-state invariant US RNA levels in the cytoplasm could be explained by increased release of HIV particles (Fig. 3B). However, we cannot exclude MATR3-dependent effects on the stability of HIV US RNA mediated by the zinc finger antiviral protein (ZAP) complex as proposed by Erazo and Goff (19).

To confirm a positive effect of f-MATR3 overexpression on HIV-1 replication, PBLs from healthy donors were transduced with the lentiviral vector expressing f-MATR3 and infected with HIV-1 NL4.3. As shown in Fig. 3F, transduction of f-MATR3 significantly increased MATR3 levels, which resulted in higher levels of viral particles released by the infected PBLs (Fig. 3G). These data complement the depletion data described above and confirm the role of MATR3 as a posttranscriptional regulator of HIV-1 RNA and viral replication.

### MATR3 depletion inhibits reactivation from latency.

**FIG 4:**
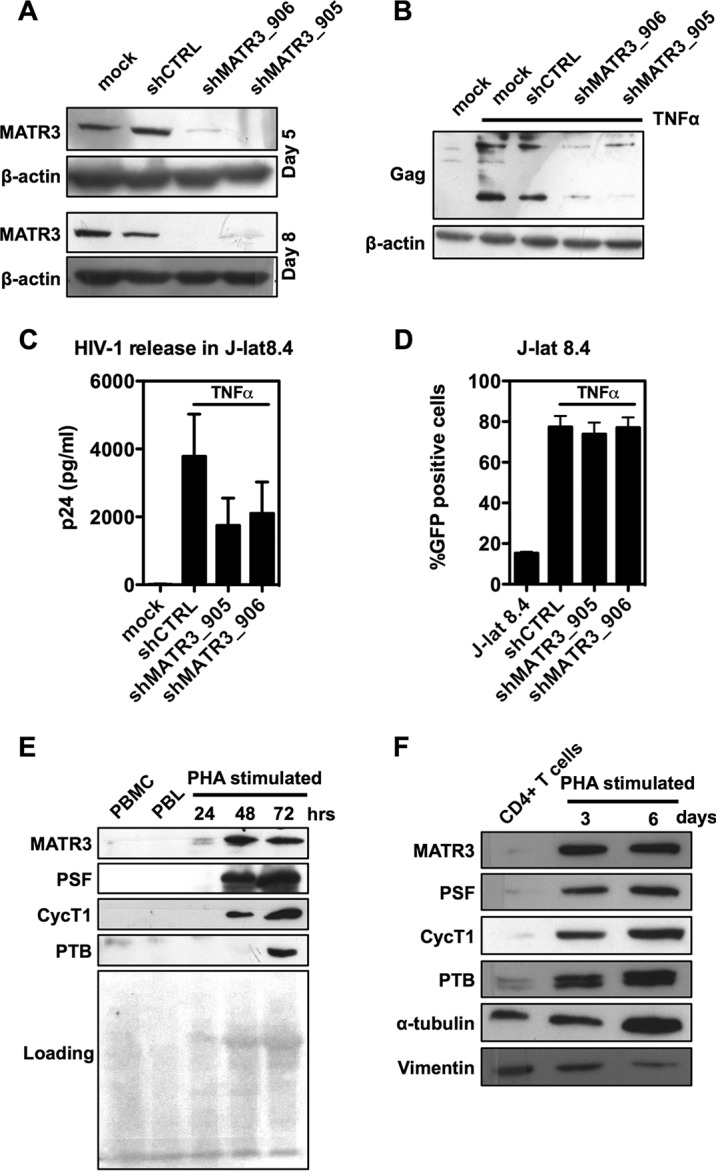
MATR3 depletion impairs full reactivation of HIV-1 from latency. (A) Efficiency of MATR3 depletion in J-lat 8.4 cells. MATR3 depletion was obtained with shMATR3 as described above (Fig. 1A). Three days after puromycin selection, MATR3-depleted cells were harvested for immunoblotting. β-Actin is the protein loading control. (B) MATR3 depletion leads to reduced HIV-1 Gag expression. J-lat 8.4 cells were transduced with shMATR3. Three days after puromycin selection, MATR3-depleted cells were stimulated with TNF-α (30 ng/ml), and 48 h postinduction, samples were collected for immunoblotting. β-Actin is the protein loading control. (C) MATR3 depletion leads to the decrease in the extracellular release of HIV-1 Gag. MATR3-depleted J-lat cells generated as described above were stimulated with TNF-α, and supernatant was harvested 48 h postinfection. Virion production in the supernatant was quantified by HIV-1 p24 ELISA. Results from three separate experiments are shown as mean values ± SD. (D) HIV-1 LTR-driven transcription is not affected by MATR3 knockdown. MATR3-depleted J-lat 8.4 cells treated as described above were analyzed for GFP expression by cytofluorimetry. The results of three independent experiments are shown as mean values ± SD. (E) Expression of MATR3 and other cofactors in PBLs. Freshly isolated lymphocytes from peripheral blood from buffy coat were stimulated with PHA/IL-2 for 3 days. Cell lysates were blotted with the indicated antibodies. Loading control is the blotting membrane stained with Ponceau S. (F) Expression of MATR3 in primary CD4^+^ T lymphocytes. Primary CD4^+^ T cells were purified by positive selection with magnetic beads and stimulated with PHA/IL-2. Three and 6 days postinduction, cell lysates were blotted with the indicated antibodies using both α-tubulin and vimentin as loading controls.

In order to investigate the role of MATR3 in reactivation from HIV-1 latency, we took advantage of the J-lat 8.4 HIV-1 latency model (20). Cells were transduced with shMATR3 to obtain a prolonged silencing of MATR3 (Fig. 4A) and then reactivated with TNF-α to induce HIV-1 transcription. As shown in Fig. 4B, cell-associated Gag protein levels were markedly reduced upon MATR3 depletion. Consistently, p24 levels in the medium were also reduced (Fig. 4C), while HIV-1 transcription, which is measured by the GFP reporter from a spliced HIV-1 mRNA, was not affected by MATR3 depletion (Fig. 4D). These experiments show that depletion of MATR3 resulted in a reduced ability of TNF-α to reactivate HIV from latency. TNF-α is a potent and unspecific inducer of HIV-1 reactivation, and the small, albeit significant, reduction of p24 release in conditions of MATR3 depletion indicates that MATR3 is involved in posttranscriptional control of HIV-1 latency, but may not be the only factor involved.

One intriguing hypothesis is that certain host cellular factors, which are essential for HIV-1 gene expression, might be limited, and/or their activities might be perturbed, in latently infected cells. Therefore, we explored the expression levels of MATR3 and other cofactors of HIV-1 transcription, such as cyclin T1, or acting posttranscriptionally (PSF and the polypyrimidine tract binding protein PTB) in primary PBLs. We observed that these factors were detectable at very low levels in quiescent PBLs, but were highly induced by PHA (Fig. 4E). The same was observed for purified primary CD4^+^ T cells (Fig. 4F). PBLs are heterogeneous and a small proportion of cells may be activated and therefore responsible for the low basal levels of MATR3 observed. More restricted populations such as CD3^+^ or CD3^+^/CD4^+^ T cells showed activation markers HLADR and CD69 in an average of 5.7% ± 1.6% and 4.9% ± 1.2% of cells, respectively. Hence, levels of MATR3 and other transcriptional and posttranscriptional cofactors required for full reactivation of HIV-1 are low in quiescent lymphocytes, possibly contributing to HIV-1 latency.

### Modulation of MATR3 levels in HIV-1-infected patients’ PBLs following treatment with latency-reversing agents.

**FIG 5:**
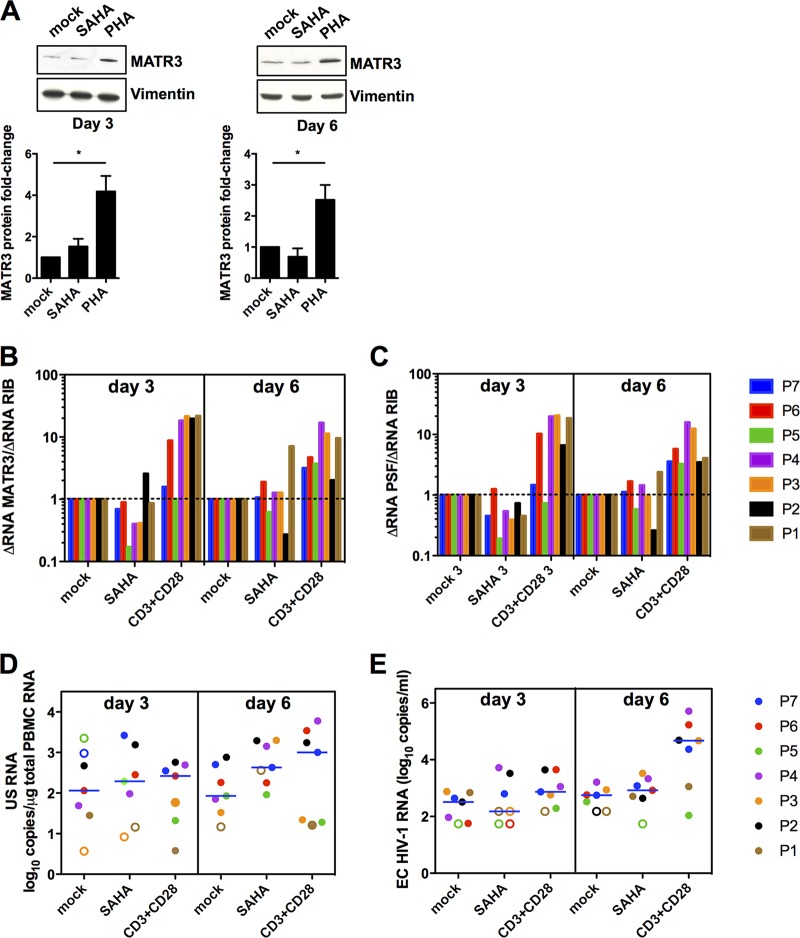
MATR3 levels in cART-treated aviremic patients’ cells correlate with low efficiency of full virus reactivation by SAHA. (A) MATR3 protein levels remain low in PBLs treated with SAHA. Uninfected PBLs were treated with 0.5 μM SAHA or 5 µg/ml PHA for three or 6 days. Whole-cell extracts were blotted for MATR3 and vimentin as loading control. Quantification was performed on independent blots from 4 different donors acquired with ImageJ and normalized to vimentin. (B) MATR3 mRNA remains low in *ex vivo* cultures of CD8^+^-depleted PBMCs from cART-treated HIV^+^ aviremic patients treated with SAHA. Latently infected, CD8^+^-depleted PBMCs purified from blood of 7 cART-treated HIV^+^ aviremic patients were activated with anti-CD3 and anti-CD28 antibodies (C+) or 0.5 µM SAHA for the indicated time points. MATR3 mRNA levels were quantified by RT-qPCR and reported as fold change compared to mock treatments per time point, normalized to the changes in total cellular RNA (dotted line). Data sets were analyzed using one-sample Wilcoxon tests to compare the normalized values in SAHA and CD3/CD28 columns to 1. *P* < 0.05 was considered statistically significant. SAHA day 3 versus mock day 3, *P* = 0.2969; CD3+CD28 day 3 versus mock day 3, *P* = 0.0313; SAHA day 6 versus mock day 6, *P* = 0.4688; CD3+CD28 day 6 versus mock day 6, *P* = 0.0156. (C) PSF mRNA remains low in *ex vivo* cultures of CD8^+^-depleted PBMCs from cART-treated HIV^+^ aviremic patients treated with SAHA. Same as in panel B above. SAHA day 3 versus mock day 3, *P* = 0.0343; CD3+CD28 day 3 versus mock day 3, *P* = 0.0313; SAHA day 6 versus mock day 6, *P* = 0.5781; CD3+CD28 day 6 versus mock day 6, *P* = 0.0156. (D) Treatment with SAHA efficiently induces HIV-1 transcription. Cell-associated unspliced HIV-1 RNA (CA-US HIV-1 RNA) extracted from *ex vivo* cultures as in panel B was quantified by RT-qPCR and expressed as HIV RNA copy numbers/µg of total cellular RNA. Open symbols indicate undetectable samples and report an estimated value calculated as 50% of a detection limit per sample. The detection limit depended on the amounts of cellular RNA and therefore differed between samples. The medians are represented. Data sets were analyzed using a paired, nonparametric Wilcoxon test. *P* < 0.05 was considered statistically significant. SAHA day 3 versus mock day 3, *P* = 0.1875; CD3+CD28 day 3 versus mock day 3, *P* = 0.3125; SAHA day 6 versus mock day 6, *P* = 0.2188; CD3+CD28 day 6 versus mock day 6, *P* = 0.2969. (E) Treatment with SAHA does not induce HIV-1 particle release. Extracellular genomic viral RNA (EC HIV-1 RNA) from patients’ cells treated as in panel B was quantified using RT-qPCR and reported as HIV RNA copy numbers/ml of plasma. Symbols and statistics as reported for panel D above. SAHA day 3 versus mock day 3, *P* = 0.6875; CD3+CD28 day 3 versus mock day 3, *P* = 0.1563; SAHA day 6 versus mock day 6, *P* = 0.2969; CD3+CD28 day 6 versus mock day 6, *P* = 0.0313.

Several latency-reversing agents have been proposed in strategies for HIV-1 eradication (1). Vorinostat (SAHA) is a well-described histone deacetylase inhibitor capable of inducing high levels of HIV-1 transcription. We observed that uninfected PBLs treated with SAHA did not show a significant increase of MATR3 protein levels, as opposed to PHA activation (Fig. 5A). To confirm this observation, we established a protocol of HIV reactivation from patients’ cells. *Ex vivo* cell cultures of CD8^+^-depleted PBMCs from 7 HIV^+^ patients on cART were treated with 0.5 µM SAHA, corresponding to the concentration found in plasma (21), or with anti-CD3 and anti-CD28 antibodies that serve as positive control of global T cell activation. We assessed the levels of MATR3 mRNA (Fig. 5B), PSF mRNA (Fig. 5C), cell-associated unspliced HIV-1 RNA (CA-US HIV-1 RNA, Fig. 5D), and extracellular HIV-1 RNA (EC HIV-1 RNA, Fig. 5E) at days 3 and 6 of treatment/stimulation. MATR3 and PSF mRNA levels did not increase upon SAHA treatment, in contrast to the increases observed in TCR-stimulated cells (Fig. 5B and C). We observed that in cells following SAHA treatment, levels of CA-US HIV-1 RNA increased at day 6 and reached similar levels of increase as observed in the positive control (Fig. 5D). Importantly, levels of genomic extracellular HIV-1 RNA were not increased following SAHA treatment and were similar to levels observed in mock cells (Fig. 5E). These results suggest that SAHA induces HIV transcription but does not lead to similar effects at the level of HIV-1 production as assessed by measurement of extracellular HIV-1 RNA levels. These data are consistent with a scenario where inability of SAHA to increase the levels of MATR3 and PSF mRNA could be linked with its inability to fully reactivate HIV-1.

**FIG 6:**
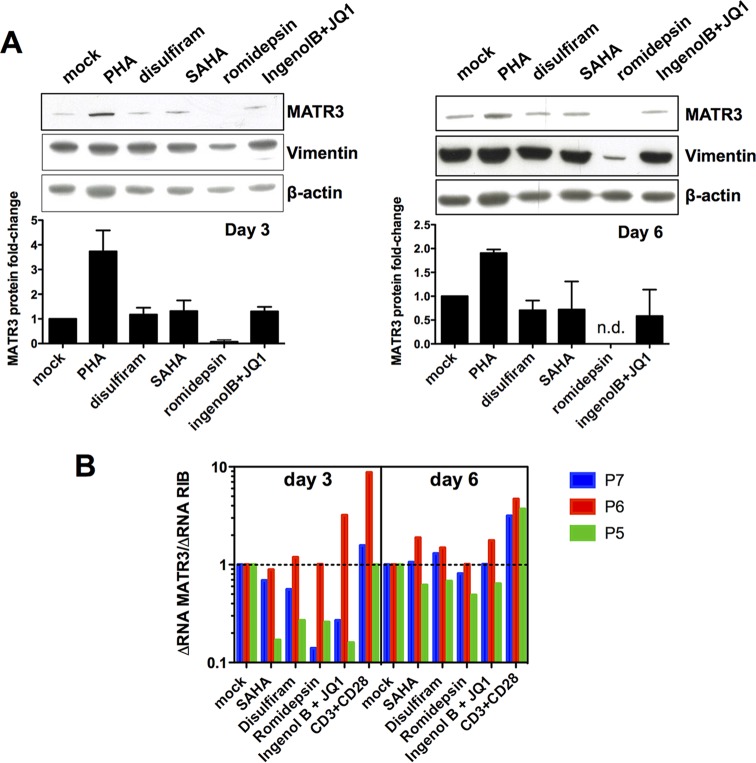
MATR3 levels in cART-treated aviremic patients’ cells correlate with low efficiency of full virus reactivation by LRAs. (A) Treatment with LRAs did not change MATR3 protein levels. Healthy donors’ uninfected PBLs treated as described in Fig. 5B were stimulated for three or 6 days with 5 µg/ml PHA, 0.5 µM SAHA, 0.5 µM disulfiram, or 0.0175 µM romidepsin or with a combined treatment of 10 nM IngenolB with 0.5 µM JQ1. Whole-cell extracts were blotted for MATR3 and vimentin as loading control. Quantification was performed on independent blots from 3 different donors acquired with ImageJ and normalized to vimentin. (B) Treatment with LRAs did not change MATR3 mRNA levels. *Ex vivo* cell cultures as in Fig. 5B were treated with LRAs as indicated above for Fig. 5E. MATR3 mRNA levels were quantified by RT-qPCR and reported as fold change compared to mock treatments per time point, normalized to the changes in total cellular RNA (dotted line). Data from the 3 patients were analyzed using the one-sample Student *t* test with significance at *P* < 0.05. SAHA day 3 versus mock day 3, *P* = 0.1917; disulfiram day 3 versus mock day 3, *P* = 0.351; romidepsin day 3 versus mock day 3, *P* = 0.1909; IngenolB + JQ1 day 3 versus mock day 3, *P* = 0.8525; CD3+CD28 day 3 versus mock day 3, 0.3819; SAHA day 6 versus mock day 6, 0.6606; disulfiram day 6 versus mock day 6, *P* = 5874; romidepsin day 6 versus mock day 6, *P* = 0.2681; IngenolB + JQ1 day 6 versus mock day 6, *P* = 0.7147; CD3+CD28 day 6 versus mock day 6, *P* = 0.0237.

In addition to SAHA, we tested several LRAs with different modes of action at concentrations found in plasma: the putative PTEN inhibitor disulfiram, the deacetylase inhibitor romidepsin, and the PKC agonist IngenolB in combination with the BET inhibitor JQ1. Protein levels of MATR3 did not increase compared to mock treatment, with the exception of romidepsin, which showed a strong decrease (Fig. 6A). However, we observed that at the concentration of romidepsin used in this experiment there is a consistent cytotoxic effect leading to a generalized decrease of protein levels, as evident looking at the vimentin loading control. To conclude this set of experiments, treatment of HIV-1-infected cells from three patients with these drugs didn’t show any significant change of MATR3 mRNA levels, which is consistent with our hypothesis (Fig. 6B).

**FIG 7:**
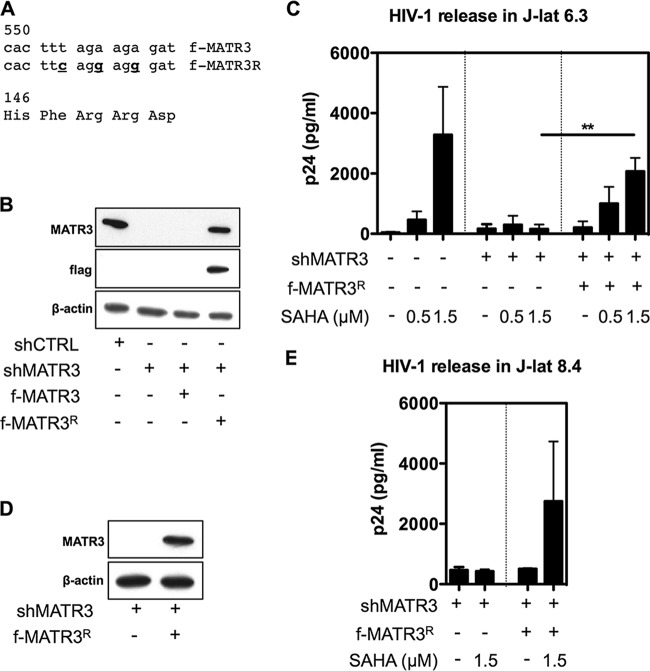
Rescue of MATR3 from J-lat-depleted cells shows dependence for HIV-1 release. (A) Design of a mutant MATR3 (f-MATR3^R^) resistant to shMATR3 depletion. The target sequence of shMATR3_905 was mutated in 3 positions without changing the amino acid composition of the protein. Inserted mutations were T552C (F147), A555G (R148), and A558G (R149). (B) Depleted endogenous MATR3 is rescued by ectopic f-MATR3^R^. J-lat 6.3 cells were transduced with shMATR3_905. Three days after puromycin selection (1 µg/ml), MATR3-depleted cells were transduced either with f-MATR3 or with the mutant f-MATR3^R^, which is the only one able to rescue MATR3 protein levels. Following incubation for 3 days in blasticidin (10 µg/ml), cells were processed for immune staining. (C) MATR3 is required for SAHA reactivation from latency in the J-lat model. J-lat 6.3 cells were left untreated or transduced as above (B) and then treated with SAHA at the concentrations indicated for 24 h. Extracellular p24 in the supernatant was quantified by HIV-1 p24 ELISA. Results from three independent experiments are shown as mean values ± SD. (D) Depleted endogenous MATR3 is rescued by ectopic f-MATR3^R^. J-lat 8.4 cells were transduced with shMATR3_905. Three days after puromycin selection, MATR3-depleted cells were transduced with f-MATR3^R^. Following incubation for 3 days, cells were processed for immune staining. (E) MATR3 is required for SAHA reactivation from latency in the J-lat model. J-lat 8.4 cells were transduced as above (D) and then treated with SAHA for 24 h. Extracellular p24 in the supernatant was quantified by HIV-1 p24 ELISA. Average results from two independent experiments are shown.

Results above showed that LRAs did not upregulate MATR3 in quiescent T cells, and we propose that this could be related to their inability to fully reactivate HIV-1 from latency. To establish a direct link between MATR3 depletion and reactivation from latency, we returned to the J-lat model of HIV-1 latency. Endogenous MATR3 is highly expressed in this model of latency, which could be depleted by shMATR3 treatment. As shown in Fig. 7C, SAHA induced p24 release in J-lat cells, while shMATR3 treatment completely abolished the effect of the drug on particle release. This defect was efficiently rescued by ectopic expression of a MATR3 variant engineered to be resistant to shRNA activity in both J-lat 6.3 and J-lat 8.4 clones (Fig. 7). By this approach we could mimic a MATR3-limiting cellular environment and address the question of its role in the posttranscriptional regulation in the context of SAHA induction. This experiment provides a direct evidence of the role of MATR3 in the posttranscriptional regulation of HIV-1 latency.

## DISCUSSION

MATR3 is an RNA-binding component of the nuclear matrix involved in diverse processes, including the response to DNA damage (22), mRNA stability (23), RNA splicing (24), and nuclear retention of hyperedited RNA (25). Mutations in MATR3 have also been linked to familial amyotrophic lateral sclerosis, adding to a list of mutations in many proteins that function in RNA processing associated with this disease (26). MATR3 has been involved as a modulator of ZAP activity in the restriction of retroviruses (19) and as a target for phosphorylation promoted by the alphaherpesvirus US3/ORF66 kinases (27). Perhaps the best-studied role for MATR3 in a virus life cycle is during HIV-1 infection. MATR3 was shown to act as a Rev cofactor and to promote the accumulation of HIV-1 unspliced and partially spliced transcripts in the cytoplasm (15, 16). We also proposed that PSF commits nascent HIV pre-mRNA to MATR3 in the insoluble, nuclear-matrix-containing fraction of the nucleus. This pathway of RRE-containing viral RNA maintenance is targeted by the Rev protein. Rev binds the nascent viral pre-mRNA to redirect it to nuclear export through a still poorly characterized mechanism that also involves MATR3 (18, 28). However, despite the definition of the role of MATR3 in the nuclear export of viral RNA, we didn’t have an understanding of the physiological role of MATR3 during viral replication and reactivation from latency. Therefore, in this work we wished to establish the role of MATR3 in acute HIV-1 infection of CD4^+^ T lymphocytes and in reactivation from latency following LRA treatment in HIV^+^ aviremic patients’ cells. First, we defined an experimental setup to modulate MATR3 levels efficiently in Jurkat cells and primary lymphocytes. Then, we demonstrated that HIV replication was significantly reduced when MATR3 was depleted, while it was enhanced when MATR3 was overexpressed. We could confirm that MATR3 acted posttranscriptionally, at the level of nuclear export of RRE-containing transcripts during acute infection of lymphocytes.

We then turned to study the effect of MATR3 modulation in a classical model of HIV latency based on Jurkat cells carrying an integrated provirus. J-lat cells had reduced levels of Gag expression when depleted of MATR3, despite full transcriptional reactivation following TNF-α stimulation. Intriguingly, quiescent PBLs showed low levels of MATR3 and PSF expression, which could be readily increased by stimulation with a potent lectin like PHA. Well-known LRAs such as SAHA, disulfiram, romidepsin, or IngenolB+JQ1 were unable to induce MATR3 levels from quiescent PBLs. Furthermore, *ex vivo* cell cultures of CD8^+^-depleted PBMCs from seven HIV^+^ patients under cART showed low levels of MATR3 and PSF that could be stimulated by anti-CD3 and anti-CD28 antibodies, but not by LRA treatments. Indeed, in this *ex vivo* model, LRAs such as SAHA (Vorinostat) were able to activate viral transcription but failed to fully express HIV-1. Thus, it is very likely that there might be a scenario in which reactivation by LRAs could be restricted because of limiting levels of MATR3 and PSF, as well as other cellular factors.

These data are consistent with the reports by other investigators showing that certain LRAs, notwithstanding robust induction of viral transcription, failed to effectively enhance virus production (29–31). These observations strongly suggest the importance of posttranscriptional blocks as one additional mechanism leading to HIV latency that needs to be relieved in order to purge the viral reservoir. Recent reports are highlighting this long-overlooked aspect of HIV reactivation from latency. Indeed, HIV latency could be redefined to include cells in the reservoir with a silent provirus, but also cells with a transcriptionally competent virus that are unable to fully process and translate viral RNAs. Since only fully replicating HIV is responsible for virus rebound, but also required for purging the reservoir by the immune system in any “shock and kill” approach, new tools are required to measure the reservoir accordingly (32, 33).

Although we were able to show direct evidence of the role of MATR3 in the posttranscriptional regulation of HIV-1 latency in the J-lat model, it would be important to investigate its role in primary lymphocytes. Several cellular models have been proposed to recapitulate HIV latency in primary lymphocytes *in vitro* (34, 35). Most of them include a step of T cell activation before infection and genes such as those coding for MATR3 and PSF could remain expressed at sublimiting levels also when the HIV-1 provirus returns to the silent state. This could be directly observed from the analysis of expressed genes in the well-described model of HIV latency in CD4^+^ T cells developed by the Karn laboratory, which can be queried from the open access interactive web resource of the Telenti laboratory at http://litchi.labtelenti.org (30, 36). Alternative strategies include the establishment of HIV latency by direct infection of resting T cells (37, 38). However, we were not successful in modulating MATR3 levels following protocols that increase quiescent T cell permissivity by treatment with virus-like particles containing Vpx to degrade the restriction factor SAMHD1 (39, 40). Notwithstanding technical difficulties, posttranscriptional regulation of HIV latency could still be a multifactorial process targeting steps of transcription, RNA processing, nuclear export, and translation, therefore requiring modulation of more than one factor at a time (8).

We conclude that cellular factors such as MATR3 and PSF are poorly stimulated by LRA treatment *ex vivo* in HIV-1-infected patients’ cells and that this could contribute to poor performance of those LRAs. These data reinforce the need to further investigate the pathways to full virus reactivation to potentiate combination LRA treatments, including posttranscriptional targets.

## MATERIALS AND METHODS

### Cells.

Human embryonic kidney T cells (TaKaRa Bio USA no. 632273) were grown in Dulbecco’s modified Eagle’s medium (DMEM) supplemented with 10% fetal calf serum (FCS) and antibiotics. Jurkat and J-lat 8.4 cells were obtained from the NIH Research and Reference Reagent Program and were maintained in Roswell Park Memorial Institute medium (RPMI) supplemented with 10% fetal calf serum, penicillin, streptomycin, and l-glutamine.

The buffy coat from healthy donors was diluted to twice its volume with RPMI, layered onto Ficoll-Hypaque, and gently centrifuged for 2,200 rpm for 30 min. PBMCs were then collected and grown in RPMI supplemented with 10% heat-inactivated FBS and antibiotics. Nonadherent peripheral blood lymphocytes (PBLs) were collected next day, stimulated with 10% RPMI containing 5 µg/ml phytohemagglutinin (PHA) for 72 h, and subsequently maintained with 10% RPMI containing 20 U/ml interleukin-2 (IL-2). Primary CD4^+^ lymphocytes were purified from PBMCs with the Miltenyi CD4 MicroBeads by direct magnetic labeling and stimulated with PHA/IL-2 as above.

CD8^+^-depleted PBMCs used in reactivation assays were isolated from fresh whole blood of HIV^+^ patients on cART as previously described (41, 42). For each treatment, six million CD8^+^-depleted PBMCs were seeded in LGM-3 growth medium (Lonza). One day after isolation, cells were mock treated or treated with anti-CD3 and anti-CD28 antibodies as a positive control or by LRAs for 3 and 6 days. Medium was harvested at day 3 and replaced with fresh medium containing LRA when appropriate.

### Antibodies for immunoblotting and chemicals.

The following antibodies have been used in the study: MATR3 (A300-590A, Bethyl Laboratories, 1:2,000 dilution for IB), HIV-1 p55 and p24 (HIV-1 p24 [1941], sc-65462, Santa Cruz, or anti-HIV-1 p55^+^ p24^+^ p17 antibody ab63917, Abcam, 1:200). Hsp90 (catalogue number ALX-804-808, Enzo Life Sciences), PARP (catalogue number ALX-210-302, Enzo Life Sciences), β-actin HRP (Sigma, AC-15, 1:10,000), PSF (B92 Sigma P2860, 1:1,000), cyclin T1 (C-20-SC-8128, 1:200), polypyrimidine tract binding protein PTB (rabbit polyclonal produced in-house, 1:1,000), flag tag (Sigma, 1:5,000), and vimentin (Cell Signaling, 1:1,000).

Other reagents included Histopaque-1077 (10771, Sigma), Polybrene (Sigma-H9268), phytohemagglutinin (PHA L1668-5MG, Sigma), interleukin-2 (H7041, Sigma), TNF-α (T0157, Sigma), puromycin (ant-pr-1, InvivoGen), blasticidin (ant-bl-1, InvivoGen), SAHA (SML0061, Sigma), disulfiram (D2950000, Sigma), romidepsin (S3020, Selleckchem), and JQ1 (2091-1, BioVision). IngenolB was kindly donated by Luiz F. Pianowski, Kyolab/Amazônia Fitomedica-mentos, Valinhos, Sao Paulo, Brazil. Human CD3 (IMI1304) and CD28 (IMI1376) antibodies were obtained from Analis (Belgium).

### Lentivectors.

pLKO.1 lentiviral vectors harboring short hairpin RNA (shRNA) targeting MATR3 were obtained from Open Biosystems (TRCN0000074903-904-905-906-907). The pLKO.1 vector plasmid expressing scrambled shRNA (obtained from Open Biosystems) was used as control (shCTRL). Ectopic expression of MATR3 was obtained by cloning a flag-tagged MATR3 (f-MATR3) cDNA into the pWPI lentivector (kind gift from Gualtiero Alvisi, University of Padua, Italy). The construct was modified by site-directed mutagenesis introducing 3 silent mutations in the target region of sh905 to make it resistance to RNAi-mediated depletion (f-MATR3^R^; Fig. 7A has details).

Vesicular stomatitis virus glycoprotein G (VSV-G)-pseudotyped lentiviral particles (LVPs) were produced in HEK 293 T cells by cotransfecting the vector plasmids with the HIV packaging plasmid (psPAX2) and the VSV-G expression (pMD2G) plasmids (Addgene). The medium was replaced 16 h after calcium-phosphate transfection. The lentiviral vector-containing supernatants were collected 48 and 72 h posttransfection, clarified, filtered through 0.45-µm syringes, aliquoted, and further stored at −80°C until further usage.

Transduction of LVPs was obtained in the presence of 10 µg/ml Polybrene. The medium was replaced after 16 h to remove unbound virus particles. At 24 h postransduction the cells were cultured with fresh 10% RPMI containing 1 µg/ml puromycin (pLKO.1 LVPs) or 10 µg/ml blasticidin (pWPI LVPs).

### HIV-1.

Infectious HIV-1 stocks were generated by transfecting the full-length HIV-1 pNL-4.3 plasmid into HEK-293T cells using the standard calcium phosphate transfection method. HIV-1-containing supernatants were collected 48 and 72 h posttransfection, clarified, filtered through 0.45-µm filters, aliquoted, and further stored at −80°C until further usage. Viral production was quantified in the supernatants for HIV-1 p24 antigen content using the Innotest HIV antigen MAb kit (Innogenetics N.V., Ghent, Belgium).

Jurkat cells or stimulated PBLs were infected with HIV-1 pNL-4.3 virus in the presence of 10 µg/ml of Polybrene. After 4 h of incubation, cells were washed twice and then cultured in complete RPMI medium supplemented with IL-2.

### Luciferase assay.

Jurkat cells were infected with 1 µg/ml of HIV-1 pNL4.3R-E-luc pseudotyped with VSV-G envelope for 4 h. The infected cells were washed twice and further incubated at 37°C for 48 h. At 48 h postinfection, the cells were harvested and lysed in passive lysis buffer and the levels of luciferase activity were measured by the single-luciferase-reporter assay (p.j.k.) as directed by the manufacturer. For normalization, total protein concentration in each extract was determined with a Bio-Rad protein assay kit.

### Nuclear and cytoplasmic fractions.

Nuclear and cytoplasmic fractions were obtained essentially as described previously (15, 18). The cytoplasmic fraction and nuclei were subjected to RNA extraction using UPzol according to the manufacturer’s protocol (Biotechrabbit). Purity of fractions was assayed by Western blot of cytoplasmic and nuclear proteins.

For quantitative real-time PCR (qRT-PCR), RNA was treated with DNase I (Invitrogen) to remove genomic DNA contamination and used as a template to synthesize cDNA using random hexamers and MMLV reverse transcriptase (Invitrogen) according to the manufacturer’s protocol. Real-time PCR amplification was conducted in the presence of KAPA SYBR FAST Bio-Rad ReadyMix (KAPA Biosystems) and monitored on a C1000 Thermal Cycler (Bio-Rad). The viral RNA abundance was calculated relative to the GAPDH mRNA expression and shown as fold change in comparison with control samples. Results were expressed as mean plus or minus standard deviations.

### Study subjects.

Seven HIV-1-infected individuals were selected at the St-Pierre Hospital (Brussels, Belgium) on the basis of the following criteria: all volunteers were treated with cART for at least 1 year, had an undetectable plasma HIV-1 RNA level (20 copies/ml) for at least 1 year, and had a level of CD4^+^ T lymphocytes higher than 300 cells/mm^3^ of blood. Characteristics (age, CD4^+^ T cell count, CD4^+^ nadir, antiviral regimens) of patients from the St- Pierre Hospital were well documented and are presented in Table 1.

**TABLE 1:** Presentation of patient characteristics^*a*^

Patient	Code	Total HIV-1 DNA copies/10^6^ cells	Age (yr)	CD4^+^ T cell count	Nadir	Last treatments
P1	431	94	41	433	433	STB
P2	403	1,329	46	807	331	KVX RTV ATV
P3	425	184	57	359	10	3TC RTV DRV RLT ETV MVC
P4	421	317	47	16	16	KVX RTV DRV
P5	410	553	56	789	321	TRU ATV
P6	408	1,627	58	NA	25	TRU RTV DRV
P7	15	1,442	59	1,083	92	RTV DRV ETV MVC

a Characteristics (age, CD4^+^ T cell count, CD4^+^ nadir, antiviral regimens) and total HIV-1 DNA copies/million of CD8^+^-depleted PBMCs of patients from the St-Pierre Hospital are presented. NA, not available. Abbreviations: STB, elvitegravir/cobicistat/emtricitabine/tenofovir DF; KVX, Kivexa; RTV, ritonavir; ATV, atazanavir; 3TC, lamivudine; DRV, darunavir; RLT, raltegravir; ETV, etravirine; MVC, maraviroc; TRU, Truvada; ETV, etravirine.

### Ethics statement.

Ethical approval for HIV-1 patients’ cells was granted by the Human Subject Ethics Committees of the Saint-Pierre Hospital (Brussels, Belgium). All individuals enrolled in the study provided written informed consent for donating blood. Uninfected peripheral blood mononuclear cells (PBMCs) were obtained from healthy blood donors after approval by the Ethical Committee of the Azienda Ospedaliero—Universitaria Ospedali Riuniti di Trieste, Italy.

### Quantification of total HIV-1 DNA.

The total cellular DNA was extracted from patient CD8^+^-depleted PBMC *ex vivo* cultures using the QIAamp DNA minikit. The total cell-associated HIV-1 DNA was then quantified by ultrasensitive real-time PCR (Generic HIV DNA cell kit, Biocentric) according to the manufacturer’s instructions (43).

### Quantification of cell-free HIV-1 RNA in culture supernatants of patient cells.

Three and 6 days after treatment with LRAs, culture supernatants from patient CD8^+^-depleted PBMC *ex vivo* cultures were collected for RNA extraction using the QIAamp Viral RNA minikit (Qiagen). HIV-1 RNA levels were quantified using the Generic HIV Charge Virale kit (Biocentric) according to the manufacturer’s instructions (detection limits of 110 HIV-1 RNA copies/ml or 300 HIV-1 RNA copies/ml depending on tested supernatant volumes).

### Quantification of cell-associated HIV-1 RNA, MATR3, and PSF mRNA.

Total RNA was isolated from the patient CD8^+^-depleted PBMCs using the Boom isolation method (44), treated with DNase (DNA-free kit; Ambion) to remove DNA that could interfere with the quantitation, and reverse transcribed using random primers and SuperScript III reverse transcriptase (all from Invitrogen). Cell-associated HIV RNA was quantified using a qPCR assay specific for the HIV *gag* region (45). The amounts of HIV-1 RNA were normalized to total cellular inputs, which were quantified in separate qPCR assays, using the detection kit for 18S rRNA (Applied Biosystems, Foster City, CA), and were expressed as the number of copies per microgram of total RNA. MATR3 and PSF mRNAs were quantified using the same cDNA preparations and normalized to 18S rRNA. MATR3 mRNA was quantified by a qPCR assay using primers MATR3 fw_1431 (5′ TCT TGG GGG ACC AGC AGT TGG A 3′) and MATR3 rev_1530 (5′ GCT AGT TTC CAC TCT GCC TTT CTG C 3′). PSF mRNA was quantified by a qPCR assay using primers PSF fw_1359 (5′ AGC AGC AAG AAA GGC ATT TGA ACG 3′) and PSF rev_1434 (5′ CAC ATT GAC TGG ACG AGG AGT TG 3′).

### Statistics.

Typically, three independent experiments in triplicate repeats were conducted for each condition examined. Average values are shown with standard deviation and *P* values, measured with a Student *t* test. Only significant *P* values are indicated by the asterisks above the graphs (*P* < 0.01 = highly significant [**]; *P* < 0.05 = significant [*]). Data from patients were analyzed using paired, nonparametric Wilcoxon test or Student’s *t* test, as detailed in figure legends (*P* < 0.05 = significant). All tests were two-sided.
